# Facial profile attractiveness in orthodontically treated Class III malocclusion patients: a comparison of extraction and non-extraction protocols

**DOI:** 10.1590/2177-6709.31.1.e2625219.oar

**Published:** 2026-05-01

**Authors:** Maria Pia SEMINARIO, Rodrigo NAVEDA, Guilherme JANSON, Arnaldo PINZAN, Aron ALIAGA-DEL CASTILLO, Silvio Augusto BELLINI-PEREIRA

**Affiliations:** 1 University of São Paulo, Dental School, Department of Orthodontics (Bauru/SP, Brazil).; 2 SEK International University, Faculty of Health Sciences, Dentistry Program, Department of Orthodontics (Quito, Ecuador).; 3 University of Michigan, Dental School, Department of Orthodontics and Pediatric Dentistry (Ann Arbor/Michigan, USA).

**Keywords:** Angle Class III, Esthetics, Soft-tissue profile, Classe III de Angle, Estética, Perfil facial

## Abstract

**Introduction:**

The treatment of Class III malocclusion has an important impact on facial and smile esthetics.

**Objective:**

To compare the facial profile attractiveness (FPA) of Class III malocclusion patients treated with and without premolar extractions.

**Material and Methods:**

A retrospective sample of 39 Class III malocclusion patients was divided into two groups: Non-extraction group, composed of 20 patients (8 males, 12 females; mean age: 13.7 years); and the Extraction group, comprising 19 patients (9 males, 10 females; mean age: 15.0 years). Both groups were treated with orthodontic fixed appliances. Facial profile silhouettes were created from the pretreatment and posttreatment lateral cephalometric radiographs. The silhouettes were evaluated by 30 laypeople (20 males, 10 females; mean age: 32.2 years) and 29 orthodontists (13 males, 16 females; mean age: 48 years). The evaluators rated FPA using a scale ranging from 1 (least attractive) to 10 (most attractive). Intergroup comparisons of FPA between treatment protocols and evaluators were performed using Mann-Whitney tests. For intragroup comparisons of pre- and posttreatment stages, Wilcoxon tests were performed.

**Results:**

The extraction group was statistically more attractive in the pretreatment stage, when compared to the non-extraction group. Both groups showed no significant differences in the intragroup comparisons of pre- and posttreatment stages. Laypeople rated FPA significantly lower than orthodontists at both stages.

**Conclusion:**

Class III malocclusion patients treated with and without extractions presented similar FPA at the posttreatment stage. No differences were observed for the FPA in the intragroup comparisons of pre- and posttreatment stages. Orthodontists were significantly less critical than laypeople.

## RESUMO

**Introdução:**

O tratamento da má oclusão de Classe III tem um impacto importante na estética facial e do sorriso.

**Objetivo:**

Comparar a atratividade do perfil facial (APF) de pacientes com má oclusão de Classe III tratados com e sem extrações de pré-molares.

**Material e Métodos:**

Uma amostra retrospectiva de 39 pacientes com má oclusão de Classe III foi dividida em dois grupos: Grupo sem extração, composto por 20 pacientes (8 homens, 12 mulheres; idade média: 13,7 anos), e Grupo com extração, composto por 19 pacientes (9 homens, 10 mulheres; idade média: 15,0 anos). Ambos os grupos foram tratados com aparelhos ortodônticos fixos. Silhuetas do perfil facial foram criadas a partir das telerradiografias cefalométricas laterais obtidas antes e após o tratamento. As silhuetas foram avaliadas por 30 leigos (20 homens, 10 mulheres; idade média: 32,2 anos) e 29 ortodontistas (13 homens, 16 mulheres; idade média: 48 anos). Os avaliadores classificaram a APF utilizando uma escala de 1 (menos atraente) a 10 (mais atraente). As comparações intergrupos de APF entre protocolos de tratamento e avaliadores foram realizadas com testes Mann-Whitney. Para comparações intragrupo entre as fases pré- e pós-tratamento, foram utilizados testes de Wilcoxon.

**Resultados:**

O grupo com extração foi estatisticamente mais atrativo na fase pré-tratamento, quando comparado ao grupo sem extração. Os dois grupos não mostraram diferenças significativas na comparação intragrupo da APF antes e depois do tratamento. Os leigos atribuíram escores de APF significativamente menores do que os ortodontistas em ambas as fases.

**Conclusão:**

Pacientes com má oclusão de Classe III tratados com e sem extrações apresentaram APF semelhante na fase pós-tratamento. A APF não mostrou diferenças na comparação intragrupos das fases pré- e pós-tratamento. Os ortodontistas foram significativamente menos críticos do que os leigos.

## INTRODUCTION

Class III malocclusion presents a significant challenge in orthodontic treatment. It is characterized by a straight to concave facial profile due to mandibular protrusion, maxillary retrusion or a combination of both.^1^ ^,^ ^2^ The treatment of this malocclusion has an important impact on facial and smile esthetics.^3^ ^,^ ^4^ When diagnosed at an early stage, such as the beginning of mixed dentition, Class III can be treated through orthopedic interventions such as conventional maxillary protraction with a facemask, with or without rapid maxillary expansion,^5^ ^,^ ^6^ chincup therapy,^7^ ^,^ ^8^ and functional appliances.^9^ When diagnosed in the late mixed dentition, maxillary protraction associated to skeletal anchorage can be used.^10^ ^,^ ^11^

Orthopedic treatment allows to attenuate craniofacial disharmonies and improve occlusal parameters, including molar relationships, overjet, and profile esthetics.^4^ Skeletal anchorage serves as an additional therapeutic option,^10^ expanding new possibilities for the orthopedic management of Class III malocclusion during early adolescence.^10^ ^-^ ^12^ Nevertheless, following the pubertal growth phase, treatment alternatives are limited to dental compensation or orthognathic surgery, depending on esthetic requirements.^6^

Treatment approaches for adult patients with Class III malocclusion may involve fixed appliances for dentoalveolar compensation, with or without extraction protocols.^4^ ^,^ ^6^ Extraction protocols enable overjet correction by the retraction of mandibular anterior teeth and are generally indicated for patients with moderate Class III malocclusion with an anterior crossbite or edge-to-edge incisor relationship.^6^ Non-extraction protocols usually consist on the use of Class III elastics,^4^ distalization of mandibular teeth,^13^ and the multiloop Edgewise archwire technique associated with Class III elastics.^14^ Additionally, skeletal anchorage, including miniplates^15^ and mini-implants placed in the buccal shelf area,^16^ allowed the treatment of more severe Class III cases without the need for surgical intervention.

Some studies have evaluated the treatment effects of non-extraction *versus* extraction protocols in Class III malocclusion.^4^ ^,^ ^17^ ^,^ ^18^ Non-extraction patients exhibited greater improvements in the maxillomandibular relationships, compared to extraction patients.^4^ ^,^ ^18^ In terms of dental effects, the extraction groups demonstrated significantly greater mandibular incisor retrusion than the non-extraction groups.^4^ ^,^ ^17^ ^,^ ^18^ Overall, both treatment protocols contributed to an overjet increase.^4^

In contemporary orthodontic practice, esthetics standards are regarded as critical determinants of social acceptance and self-esteem. Thus, there has been a growing interest in facial beauty and attractiveness.^19^ ^,^ ^20^ Furthermore, it is essential to consider how alterations in the facial profile are perceived when selecting a treatment protocol.^21^ ^,^ ^22^ In this regard, several studies investigated the influence of extraction and non-extraction treatments on facial esthetics and attractiveness in Class I and II malocclusions.^22^ ^-^ ^25^ However, limited research has been conducted on Class III malocclusion patients.^3^ ^,^ ^26^ ^,^ ^27^ Therefore, the aim of the present study was to compare the profile attractiveness in Class III malocclusion patients treated with and without extractions at the initial and final stages of orthodontic treatment.

## MATERIAL AND METHODS

### PARTICIPANTS

This study was approved by the Ethics in Research Committee of Bauru Dental School, University of São Paulo (#22075319.6.0000.5417). The sample size calculation was based on a standard deviation for UL-Pog’Cm of 1.4mm^22^ with a minimum intergroup difference of 1.25mm. A significance level of 0.05 and test power of 80% were adopted. The minimum sample size for each group was 18 patients.

The sample of this retrospective study was selected from the archives of the Bauru Dental School, University of São Paulo. The inclusion criteria consisted of patients in the permanent dentition presenting at least a ½-cusp Class III molar relationship on at least one side; treated with orthodontic camouflage with or without premolar extractions; and initial and final records in good condition. Patients were required to exhibit a maximum anterior crossbite of 2mm, and to have undergone treatment exclusively with fixed orthodontic appliances. Sample collection did not discriminate Class III malocclusion etiology, and patients with mandibular protrusion and/or maxillary retrusion were considered. Exclusion criteria comprised patients presenting congenital or syndromic craniofacial anomalies; any history of previous orthopedic or orthodontic treatment that could alter skeletal or soft-tissue characteristics; tooth agenesis (except third molars), supernumerary or impacted teeth requiring surgical intervention; loss of anterior teeth before or during treatment; and poor-quality lateral cephalometric radiographs that did not allow accurate visualization or tracing of soft-tissue contours.

The non-extraction group (NE group) comprised 20 patients (8 males; 12 females) with an initial mean age of 13.7 ± 2.53 years and overjet of 0.70 ± 1.51 mm. The extraction group (E group) comprised 19 patients (9 male; 10 female) with an initial mean age of 15.0 ± 3.51 years and overjet of 1.10 ± 1.51 mm treated with a four-premolar extraction protocol (Table 1).

**Table 1:** Comparability between non-extraction ( NE ) and extraction ( E ) groups regarding pretreatment and posttreatment ages, treatment time, sex, Class III malocclusion severity distributions and overjet ( t tests and Chi-square tests ).

Variables	NE group (n=20)	E group (n=19)	P value
Age (years)			
Pretreatment age	13.7 ± 2.53	15.0 ± 3.51	0.171^T^
Posttreatment age	16.9 ± 2.71	18.2 ± 3.60	0.167^T^
Treatment Time	3.16 ± 1.45	3.27 ± 1.26	0.789^T^
Sex			
Female	12	10	0.688^α^
Male	8	9	
Class III malocclusion severity			
½-cusp	12	10	0.540^α^
–-cusp	6	6	
Full cusp	2	3	
Overjet	0.70 ± 1.51 mm	1.10 ± 1.51 mm	0.406^T^

^T^ t tests; ^α^ Chi-square test.

### TREATMENT

The mechanics used for Class III compensatory treatment was performed with fixed Edgewise appliances, with 0.022x0.028-in conventional brackets. Leveling and alignment was performed using 0.014-in, 0.016-in, 0.018-in NiTi archwires, followed by 0.018-in, 0.020-in, and 0.018x0.025-in or 0.019x0.025-in stainless steel archwires. Anteroposterior correction was obtained with heavy 3/16” Class III intermaxillary elastics beginning in the 0.018x0.025-in stainless steel archwire. In the extraction treatment, a similar archwire sequence was followed; but differently, anterior teeth retraction was performed using rectangular stainless steel archwires, both with elastomeric chains. Again, heavy 3/16” Class III intermaxillary elastics were used during the retraction stage.

The pretreatment (T0) and posttreatment (T1) lateral cephalometric radiographs were scanned and digitally stored.

### FACIAL PROFILE ATTRACTIVENESS

**Figure 1:**
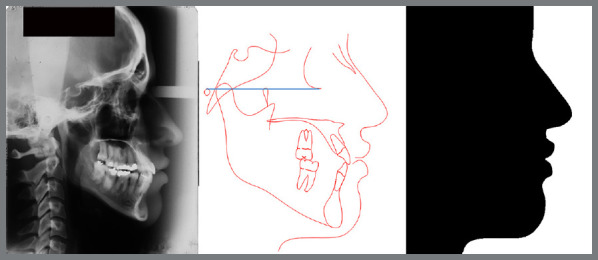
Steps performed to achieve a black shadow of the patient’s profile.

Cephalometric tracings were performed using Dolphin Imaging 11.5 software (Dolphin Imaging and Management Solutions, Chatsworth, Calif). The head position was oriented with the Frankfort plane parallel to the floor. Profile silhouette images were obtained from tracings of the soft tissue contour. The images were colored black, using Photoshop 2020 for Mac software (Adobe Systems Incorporated, San Jose, CA, USA, v21.2.0) to reproduce only a shadow of the profile. The resulting image was a black profile on a white background (Fig. 1).

Assessment of profile attractiveness was carried out using a Google^®^ form questionnaire (Google LLC, Mountain View, CA, USA) created exclusively for this research. An invitation to participate in the study was sent by e-mail and WhatsApp^®^ messenger (Facebook Corp, USA, v. 22.3.76) with a link to the form.

**Figure 2:**
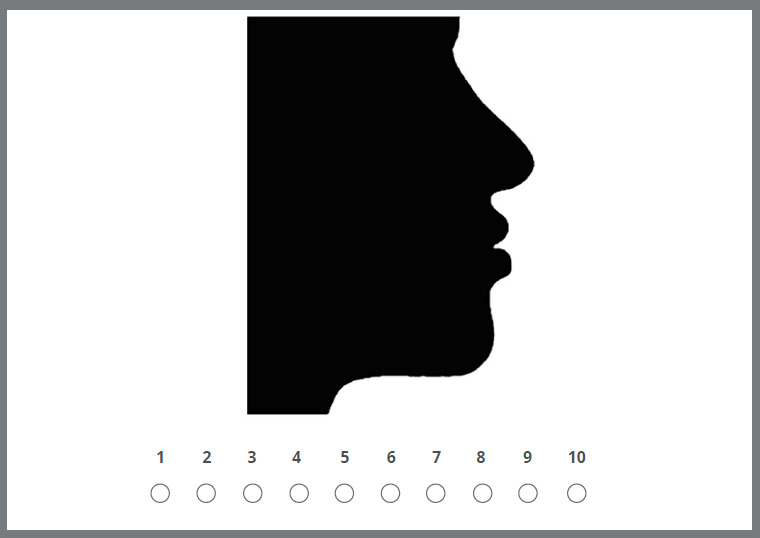
Example of silhouette photograph evaluation.

The subjective evaluation of facial profile attractiveness was scored on a 10-point numerical scale, with each photograph scored from 1 (“least attractive”) to 10 (“most attractive”; Fig. 2). Evaluators could view the images as long as they wished and change scores before submitting the form.

To determine an adequate number of evaluators, a sample size calculation was performed considering a significance level of 0.05, 80% test power, to detect a difference of 1 point on a scale of 1 to 10 between the two groups, with a standard deviation of 1.28 points, obtained from a previous study.^22^ Although 27 evaluators were necessary, 29 orthodontists (13 males; 16 females) with a mean age of 48.0 ± 9.14 years and 30 laypeople (20 males; 10 females) with a mean age of 32.2 ± 9.13 years were selected. Laypeople were defined as individuals without formal education in Dentistry or dental hygiene, selected from an e-mail list of patients receiving orthodontic treatment at the university where the study took place. Patients were encouraged to share the evaluation link with their friends and family members, for a broader participation of lay evaluators. Orthodontists were dental surgeons with completed graduate degrees in Orthodontics with 10 years of clinical experience, selected from the Brazilian Association of Orthodontists website.

### ERROR STUDY

After 30 days from the first evaluation, the entire sample was reevaluated by 14 evaluators (7 laypeople and 7 orthodontists), and the Intraclass Correlation Coefficient (ICC) was calculated to verify the intra-rater agreement in the numerical scale scores.^28^

### STATISTICAL ANALYSIS

Normal distribution was assessed using the Shapiro-Wilk test. To evaluate the comparability of the sample of Class III patients, comparisons regarding age, sex distribution, severity of Class III malocclusion and overjet were performed with t tests and Chi-square tests. For evaluators, intergroup comparisons regarding sex distribution and age were performed with Chi-square and Mann Whitney U tests, respectively.

Intergroup comparisons of profile attractiveness between non-extraction and extraction protocols, considering all the evaluators, was performed using Mann-Whitney tests. Intragroup comparisons of initial and final stages was performed using Wilcoxon tests. To compare the scores between orthodontists and laypeople, Mann-Whitney tests were used.

All tests were performed using Jamovi software (version1.6). Results were considered significant at P < 0.05.

## RESULTS

Excellent intra-rater agreement was observed, with ICC values of 0.91 and 0.81 for orthodontists and laypeople, respectively.^29^

The extraction and non-extraction groups were comparable regarding pre- and posttreatment ages, treatment time, sex, Class III malocclusion severity distributions and initial overjet (Table 1). Evaluator groups were comparable regarding sex distributions, though laypeople were significantly younger than orthodontist (Table 2).

**Table 2:** Comparison between evaluator groups regarding sex distributions and age ( Chi-square and Mann-Whitney tests, respectively ).

Variables	Laypeople (n=30)	Orthodontists (n=29)	P value
Male	20	13	P = 0.365^α^
Female	10	16	
Age (years)	32.20 ± 9.13	48.00 ± 9.14	< 0.001^*^

* Statistically significant at P < 0.05.

The extraction group was significantly more attractive in the pretreatment stage, when compared to the non-extraction group (Table 3). No significant differences were observed between non-extraction and extraction groups at the final stage.

**Table 3:** Intergroup and intragroup comparisons on facial profile attractiveness at pre- and posttreatment stages between non-extraction ( NE ) and extraction ( E ) groups ( Mann-Whitney and Wilcoxon paired tests, respectively ).

Facial profile attractiveness	NE Group	E Group	P value
	Median (IQR)	Median (IQR)	
Pretreatment (T0)	3.92 (1.64)	4.83 (1.26)	0.004^*T^
Posttreatment (T1)	4.00 (2.22)	4.32 (1.92)	0.600^T^
*P*	0.240^α^	0.249^α^	

*Statistically significant at P < 0.05, ^T^ Mann-Whitney; ^α^ Wilcoxon paired test. IQR = Interquartile Range.

Orthodontists assigned significantly higher attractiveness scores than laypeople at both initial and final stages (Table 4).

**Table 4:** Intergroup comparison of facial profile attractiveness at initial and final stages between orthodontists and laypeople ( Mann-Whitney tests ).

Variables	Laypeople	Orthodontists	P value
	Median (IQR)	Median (IQR)	
Facial profile attractiveness (T0)	3.65 (2.39)	4.15 (2.02)	0.002^*^
Facial profile attractiveness (T1)	3.77 (2.28)	5.05 (1.90)	< 0.001^*^

*Statistically significant at P < 0.05. IQR = Interquartile Range.

## DISCUSSION

The impact of Class III malocclusion on facial profile and the increasing interest of patients in esthetics have changed the goals of orthodontic treatment. Nowadays, the main objective is not just an ideal occlusion, but also better facial harmony.^3^ ^,^ ^27^ Therefore, it is important to understand the patients’ expectations of treatment.

The selected sample consisted of Class III malocclusion patients with comparable ages, sex, malocclusion severity and initial overjet (Table 1). Anteroposterior molar relationship severity and initial overjet are important because it provides information about the needed amount of mandibular anterior teeth retraction.^27^ ^,^ ^30^ ^,^ ^31^ More severe Class III patients are more likely to be treated with orthognathic surgery.^30^

To the authors’ knowledge, this is the first study comparing the facial profile attractiveness of Class III patients treated with and without extractions. Cephalometric and occlusal changes in Class III patients have been previously studied.^4^ ^,^ ^18^ ^,^ ^32^ However, the impact of these two approaches on facial profile attractiveness has not been reported.

Silhouette evaluation is a reliable and reproducible method to assess the profile attractiveness.^3^ ^,^ ^22^ ^,^ ^25^ ^,^ ^27^ ^,^ ^33^ In this study, the method allowed the standardization of the head position with the Frankfort plane parallel to the floor and eliminated characteristics such as skin color, eye color, hair and imperfections that could influence evaluators’ responses.^22^ ^,^ ^25^

With all images previously randomized, the evaluation of facial attractiveness was conducted thought a Google form questionnaire.^20^ All evaluators were instructed to observe the images carefully as many times as they wanted, with no time limit.

The higher facial profile attractiveness observed in the pretreatment for the extraction group might be associated with the slightly better initial overjet. However, camouflage treatment of Class III malocclusion, with or without extraction, produces maxillary incisors proclination, mandibular incisors retrusion, and downward and backward rotation of the mandible.^4^ ^,^ ^18^ ^,^ ^27^ It has been reported that, despite the occlusal correction, the impact of treatment on soft tissue is discrete and similar to growth changes.^4^ This was corroborated by the results of the present study, where no significant differences were found on the profile attractiveness between the two protocols after treatment. The finding that both extraction and non-extraction protocols yielded comparable posttreatment profile attractiveness highlights that the esthetic impact of compensatory treatment in Class III patients may be more limited than commonly perceived.

Studies evaluating Class II patients profile attractiveness usually report that orthodontists are esthetically more demanding than laypeople.^20^ ^,^ ^22^ ^,^ ^33^ ^-^ ^35^ Contrarily, the results of this study showed that laypeople were significantly more critical than orthodontists regarding the profile attractiveness of Class III patients (Table 4). Similar findings were described by Bou Wadi et al.,^3^ whose reported that laypeople were more critical than orthodontists when evaluating the facial attractiveness of Class III patients treated with camouflage and orthognathic surgery. Reis et al.^36^ compared the smile attractiveness of Class III patients treated with camouflage and orthognathic surgery, and found that laypeople were also more critical than orthodontists. It could be speculated that this probably occurs because orthodontists consider the type of malocclusion and treatment complexity during the evaluations of attractiveness. Thus, the authors speculate that this difference is related to the orthodontists’ knowledge of the difficulty of treating this type of malocclusion.

Treatment planning for Class III malocclusion patients should focus on the patients’ major complaints and the impact on the facial profile.^3^ ^,^ ^27^ Extraction treatment protocols involve more mandibular incisor retrusion than non-extraction treatment.^4^ ^,^ ^18^ Based on the results of this study, profile attractiveness was similar between both non-extraction and extraction groups. In addition, the significant improvement observed only in the extraction group might not be clinically significant. Thus, both protocols can achieve acceptable and similar facial results.

Some limitations of this study are related to the retrospective design. However, this is the first study comparing these treatment protocols using this methodology. Additionally, the etiology of Class III malocclusion was not considered in the present study, indicating the need for future studies with greater samples that allow subgroup analyses based on etiology, to confirm and extend the present findings. It should be also considered that facial esthetic perception is highly subjective and can be influenced by several variables, including the specific socioeconomic environment of the evaluators.^19^ ^,^ ^37^ Therefore, extrapolation of the results should be done with caution.

## CONCLUSIONS

- Class III malocclusion patients treated with and without extraction protocols showed similar facial profile attractiveness at posttreatment stage.
- Facial profile attractiveness showed no differences in the intragroup comparisons of pre- and posttreatment stages.
- Orthodontists were significantly less critical than laypeople in evaluating the facial profile attractiveness at both pre- and posttreatment stages.
